# Evolution and dip effect of boundary spatial morphology of top-coal limit equilibrium zone in steeply dipping coal seam

**DOI:** 10.1038/s41598-026-43091-w

**Published:** 2026-03-05

**Authors:** Xiaobo Wu, Xiaolou Chi, Ding Lang, Yongping Wu, Zixin Zhang, Shuaiming Chen

**Affiliations:** 1 College of Energy and Mining Engineering, Xi’an University of Science and Technology, Xi’an, 710054 China; 2 Key Laboratory of Western Mines and Hazard Prevention, Ministry of Education of China, Xi’an, 710054 China; 3 School of Mining Engineering, Anhui University of Science and Technology, Huainan, 232001 China

**Keywords:** Steeply dipping coal seam, Fully mechanized top-coal caving, Top-coal limit equilibrium zone, “Support-surrounding rock” system, Dip effect, Energy science and technology, Engineering, Solid Earth sciences

## Abstract

As the sole medium between the support and the roof, the mechanical state of top-coal governs the stability of the “support-surrounding rock” system in steeply dipping coal seam. Therefore, accurately predicting the evolution of the top-coal failure boundary morphology is essential for the stability control of the system. Based on the fully mechanized caving face at Changshanzi Coal Mine as the engineering background. A quantitative analysis of the evolution of the boundary spatial morphology of the top-coal limit equilibrium zone and its dip effect was achieved, and the instability mechanism of the “support-surrounding rock” system in steeply dipping coal seam was revealed. The evolution of the boundary spatial morphology in the top-coal limit equilibrium zone can be divided into three stages: the initial stage, the formation stage of the “asymmetric arc-shaped ribbon-like curved surface,” and the stable stage. The evolution exhibits asymmetry and sequential nature. (1) As the working face advances, the asymmetry of the boundary spatial morphology along the dip direction gradually intensifies. (2) Along the dip direction of the working face, the evolution occurs sequentially from the lower, lower-middle, upper, to the upper-middle sections. Along the strike direction, the evolution proceeds in a top-down order. As the dip angle of the coal seam increases, the evolution of the boundary spatial morphology accelerates, accompanied by an increase in failure depth and a more pronounced asymmetry in the boundary morphology. This offers solid theoretical support for guiding safe and efficient production in similar mine working faces.

## Introduction

The formation of coal seams is governed by specific sedimentary environments and depositional processes. For steeply dipping coal seam (SDCS), their development is further influenced by tectonic movements, resulting in complex structural controls that contribute to intricate seam characteristics and stress environments. Additionally, the coal quality of SDCS tends to vary, with the majority being classified as scarce resources^1,2^. During the mining of thick, SDCSs, the dip angle effect significantly increases mining difficulty and complicates strata movement and control. As the dip angle rises, the complexity of control grows exponentially^3^. As the direct medium for transmitting overlying strata pressure, the dynamically changing mechanical state of top-coal is essential for maintaining the balance of the “support-surrounding rock” system and controlling coal caving effectiveness. Therefore, accurately understanding and regulating the mechanical state of top-coal is critical to ensuring safe and efficient mining in fully mechanized top-coal caving faces.

The progressive failure mechanism of top-coal under mining pressure is a key scientific issue in the study of strata control around the working face support and the cavability of top-coal^4,5^. In terms of mining-induced stress, the distribution of principal stresses in the fully mechanized caving face of nearly horizontal coal seams is mainly symmetrical, generally exhibiting a pattern of higher values in the middle and lower values on both sides. Along the strike direction, the stress curve is predominantly unimodal, and the evolution process remains relatively stable as the advancing distance increases^6–8^. In steeply dippingcoal seams, the mining-induced stress exhibits zonal characteristics due to the gravitational-dip effect, and the failure process of the top-coal is similarly zoned, with varying evolution processes of mining-induced stress^9^. As the dip angle of the coal seam increases, the stress evolution in the stope demonstrates both regional and sequential characteristics^10^.

The control of the “support-surrounding rock” system mainly involves the following aspects: the disturbance effect of top-coal conditions on the interaction between the support and surrounding rock^11^; the coupling mechanism between large-mining-height hydraulic supports and surrounding rock^12^; the development of intelligent remote top-coal caving control technology for ultra-thick coal seams based on intelligent mining equipment and hydraulic support systems^13,14^; and the analysis of support stability control in variable-angle fully mechanized top-coal caving faces in SDCS^15^.

Regarding the cavability of top-coal, research has primarily focused on the drawing theory of top-coal as granular media. Based on the drawing law of top-coal in fully mechanized top-coal caving mining, the BBR research framework has been established^16^, and the morphology of the coal-rock interface has been analyzed^17^. Major factors affecting the recovery rate of top-coal include top-coal thickness, shearer cutting height, and top-coal fracture angle^18^. Additionally, the influence of working face inclination on the drawing body of top- coal, the coal-rock interface, and the recovery rate of top-coal has been examined^19^.

Research on the progressive failure mechanism of top-coal has primarily focused on the damage characterization of the quasi-continuous gradual deterioration process of top-coal under mining disturbance^20,21^, the control mechanism of mining-induced stress on top-coal fractures^22^, and the spatiotemporal localization of typical mechanical responses of top-coal at the macroscopic stope scale^23^. Additionally, it has revealed the damage deterioration mechanisms and extent of top-coal under varying external loads along the dip direction of the working face^24^.

Research on the fragmentation mechanism of top-coal in fully mechanized top-coal caving faces of steeply dipping coal seam remains relatively limited. Influenced by the dip angle, the deterioration process and fragmentation evolution of top-coal along the dip direction under mining-induced effects are not well understood. There is also a scarcity of studies on the boundary evolution of the mechanical state of top-coal and its dip-related effects. Quantifying the evolution of the boundary spatial morphological of the top-coal limit equilibrium zone (TLEZ) and revealing its dip angle effect can provide robust theoretical guidance for the layout design of similar stope and for the stability control of the “support-surrounding rock” system.

## Engineering background

**Fig. 1:**
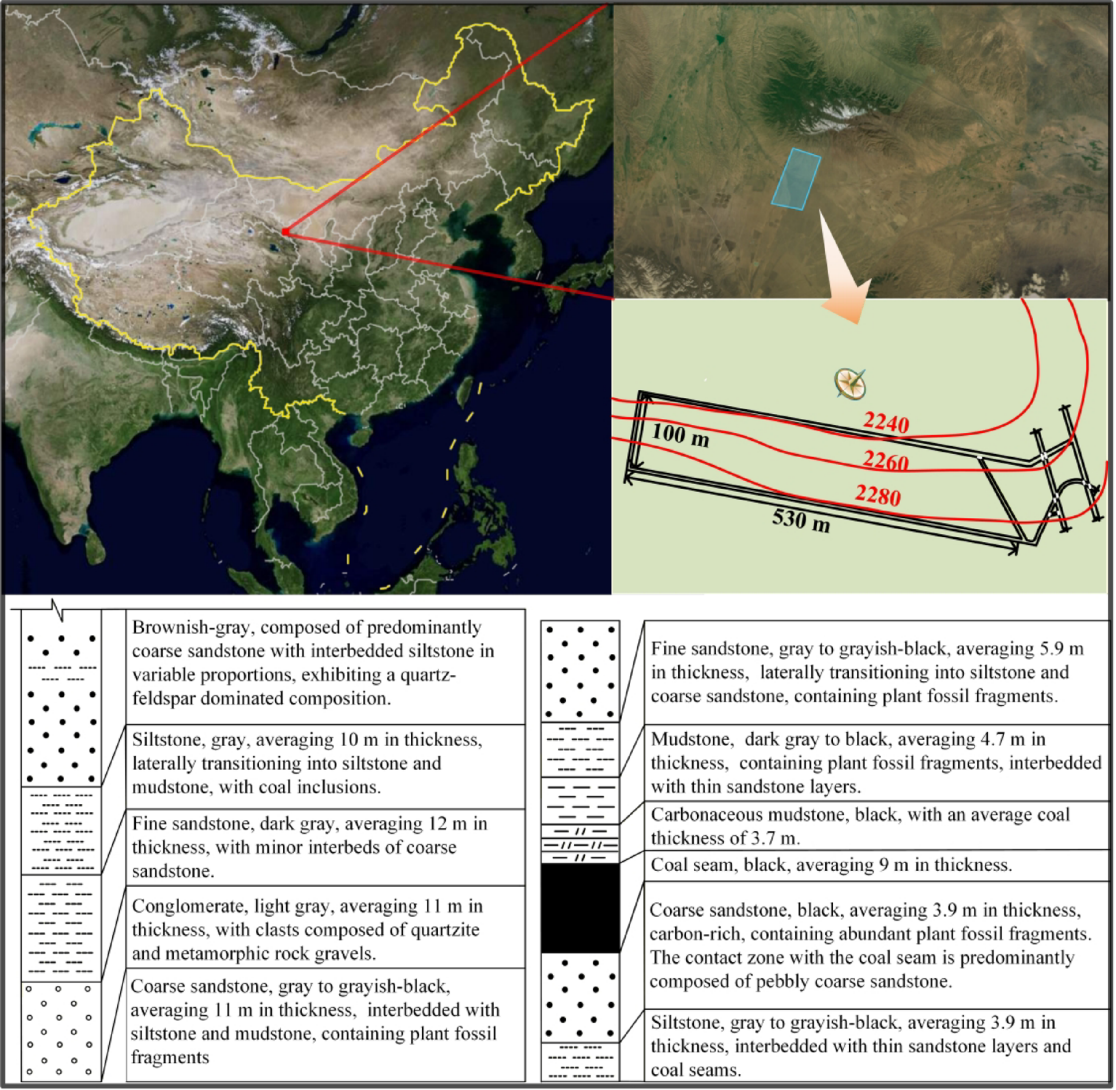
Geographical location and strata of coal mine. The base image was obtained from the Amap platform (Amap Vision Image) [Amap Software Co., Ltd., Version 16.10.0.2015, Map Approval Number: GS (2025)1807, URL: https://www.amap.com].

Changshanzi Coal Mine is located in Shandan County, Zhangye City, Gansu Province. The mine is about 5 km long from east to west, with an average width of about 2 km from north to south and an area of about 10 km². The first mining district in the mine field is District 11, which has a strike length of 1,200-2,500 m and a dip length of approximately 700 m. The coal seam has an average dip angle of 35°, a thickness ranging from 7.96 to 10.06 m, and is minable throughout the entire district. Working Face 1123 is located in the southern wing of District 11. The coal seam has an average thickness of 9 m and a dip angle of 35°. The face dimensions are 540 m along the strike and 100 m along the dip. The fully mechanized top-coal caving mining method is adopted, with a shearer cutting height of 3 m and an average top-coal thickness of 6 m. The surface above the working face consists of gently rolling hills with relatively flat terrain and no structures requiring protection. The overburden thickness ranges from approximately 300.5 m to 360.5 m, with an average burial depth of about 330 m for the working face. Detailed conditions of the roof and floor of the working face are shown in Fig. 1.

## Research methods and analytical means

### FLAC3D numerical simulation

**Fig. 2:**
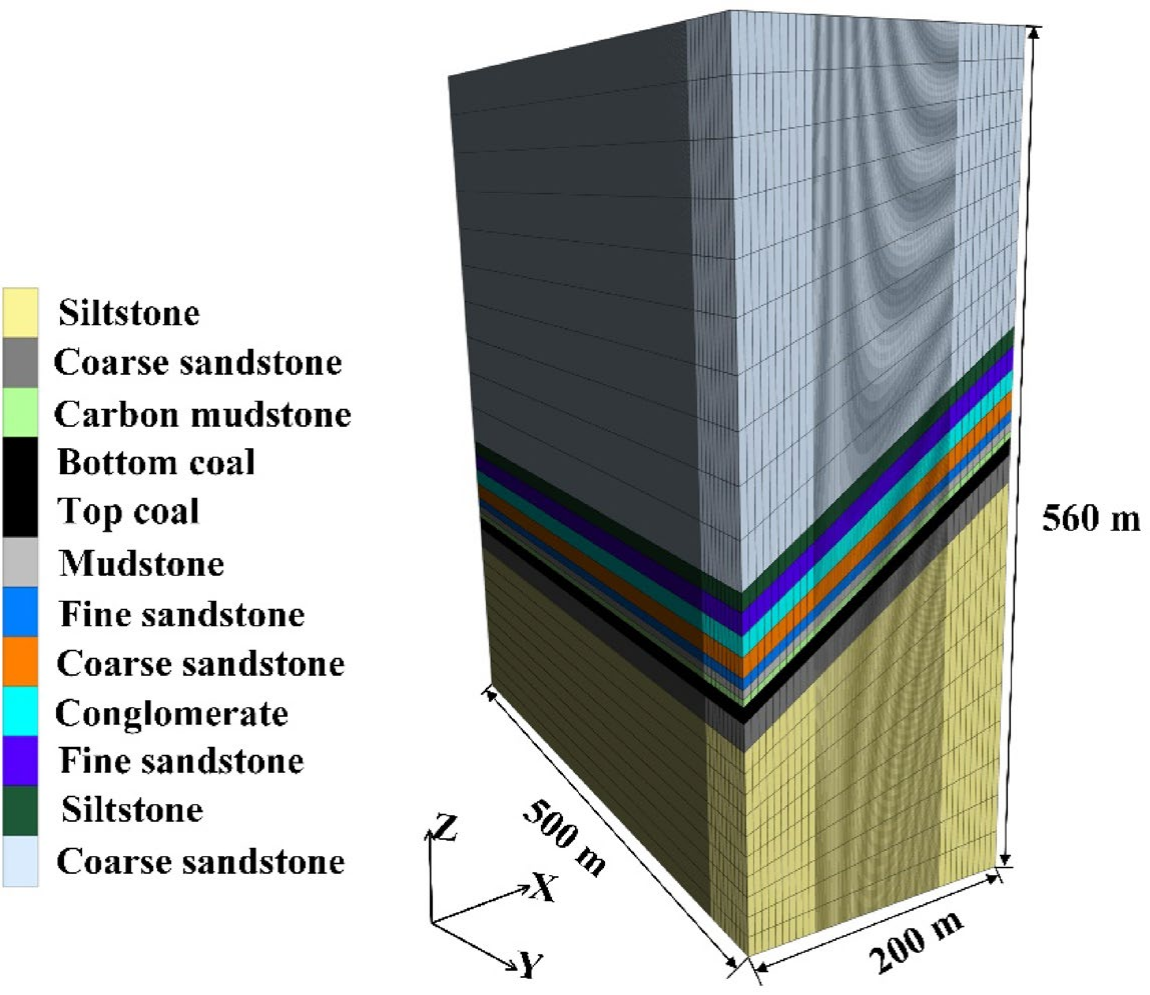
Numerical model diagram.

**Fig. 3:**
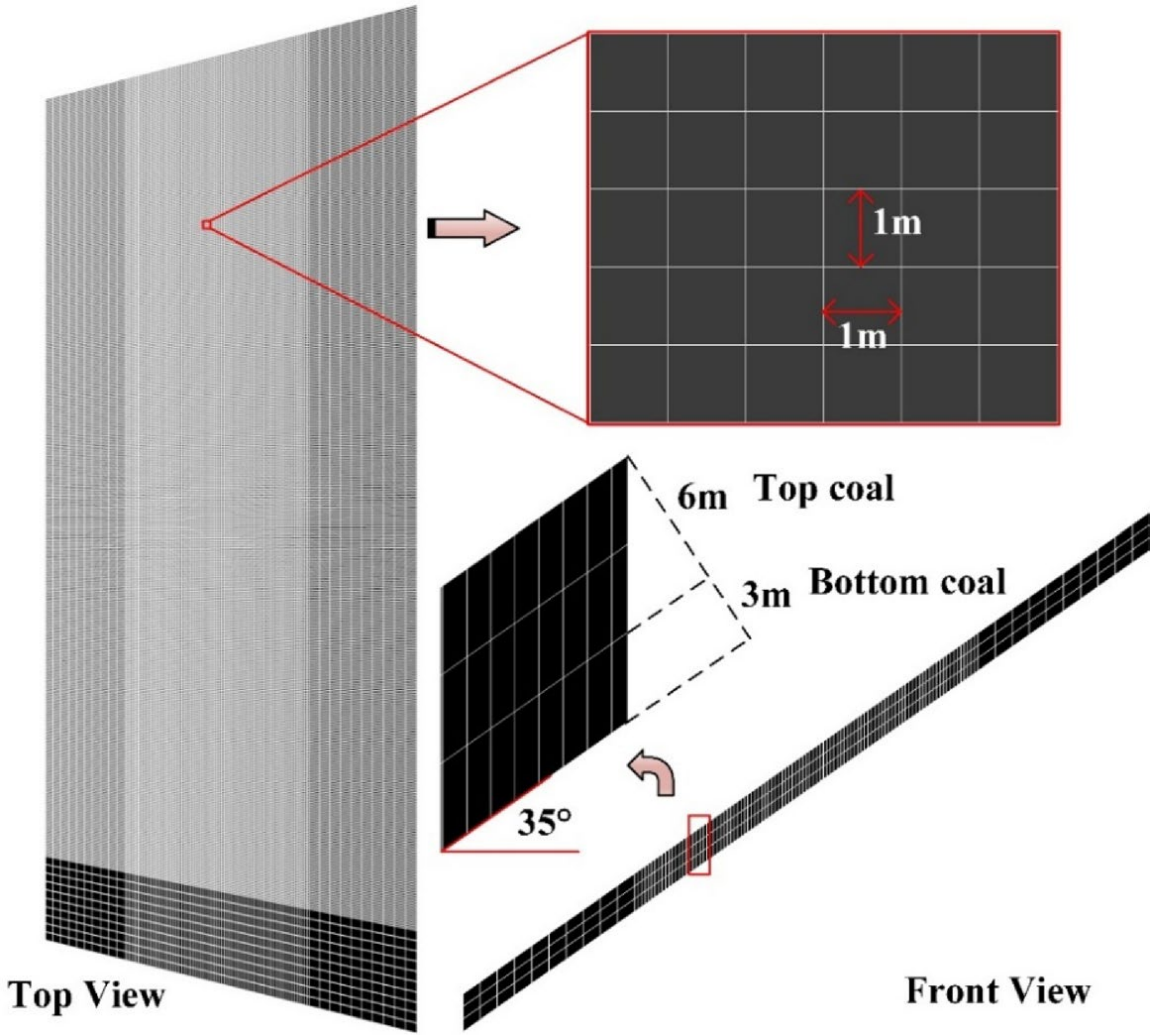
Grid division of numerical model.

A numerical model was established based on the conditions of Working Face 1123, with dimensions of 500 m (length) × 200 m (width) × 560 m (height), as shown in Fig. 2. The griding was determined according to the mechanical properties of different strata, with data provided by the coal mine. Given that the core focus of this study is top-coal, the coal seam was finely grided, as illustrated in Fig. 3, which shows the front view and top view of the seam. To improve computational accuracy and efficiency, local grid refinement was applied to the model. As seen in the top view, the central portion of the coal seam was refined, resulting in a grid size of 1 m × 1 m in that area. This refined grid facilitates later extraction of stress data and post‑processing. The front view indicates that the coal seam is divided into three layers: one layer of bottom coal and two layers of top-coal, with a dip angle of 35°.

**Fig. 4:**
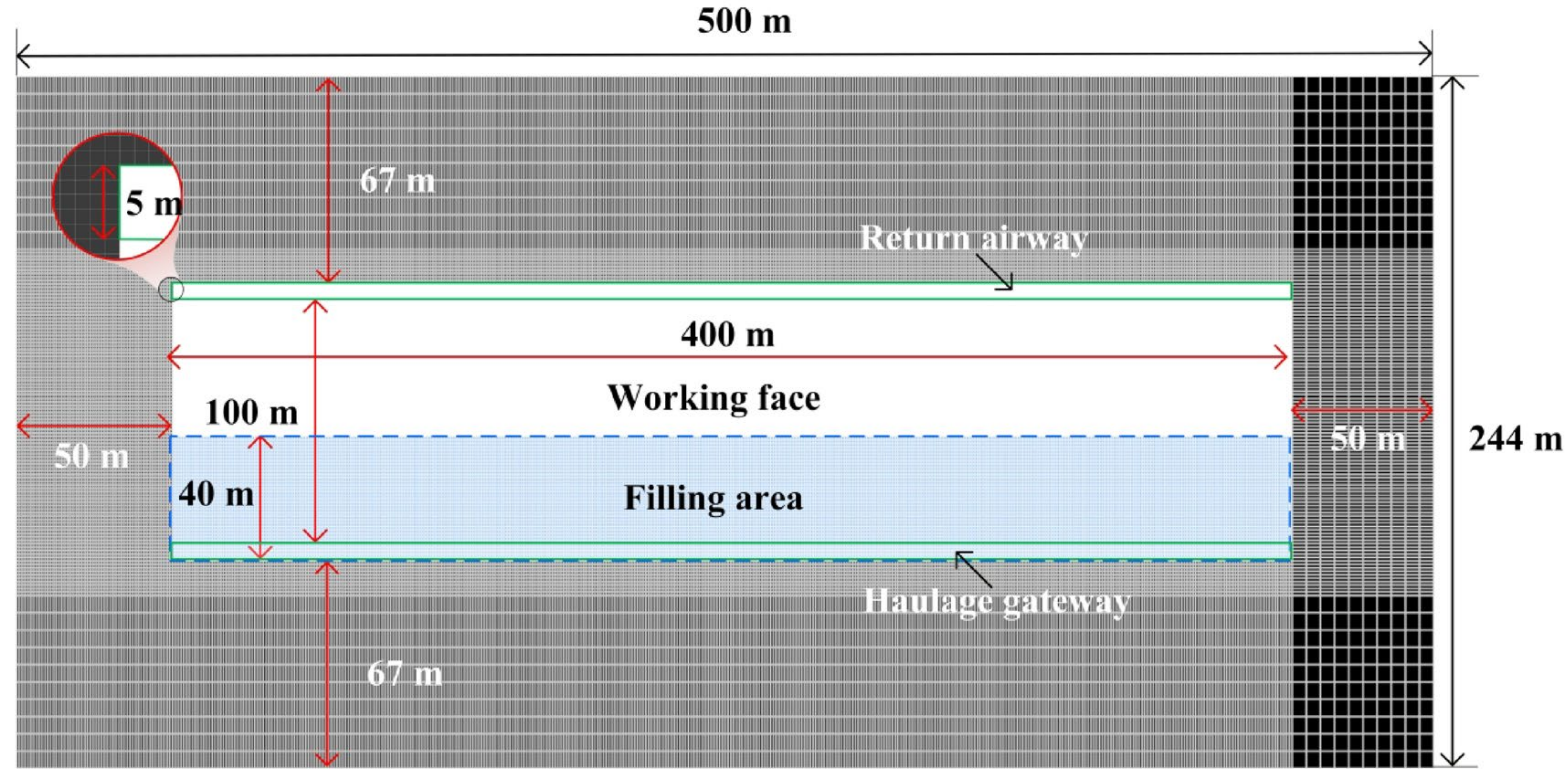
Boundary coal pillar of numerical model.

In the numerical simulation, the burial depth of the coal seam is consistent with the actual depth of 330 m. As the ground surface above the seam is relatively flat, no additional stress boundary is required on the upper boundary, which is set as a free surface. The lower boundary is assigned a fixed displacement constraint to restrict movement in all directions. The four lateral boundaries are constrained horizontally to limit transverse displacement. Additionally, the gravitational acceleration in the model is set to 9.8 m/s². In numerical simulations, boundary coal pillars are retained. This minimizes the interference from artificial boundaries on the model’s mechanical behavior. Consequently, the stress state at the model boundaries can more accurately represent true far-field conditions. This ensures that the simulation results in the core research area possess physical authenticity and engineering reference value. Therefore, careful consideration and appropriate sizing and placement of boundary coal pillars are essential. As shown in Fig. 4, which presents a horizontal cross-section along the coal seam, boundary coal pillars of 50 m are reserved on both sides along the strike direction of the working face, and 67 m on both sides along the dip direction. The distances from the coal seam to the upper and lower surfaces of the model are 330 m and 221 m, respectively. The working face length is 100 m, with an advancement distance of 400 m. The haulage roadway and the return airway have a width of 5 m. During the mining of SDCS, the collapsed roof strata tend to roll and slide. This process divides the goaf area into three zones along the dip direction: the filling compaction zone, the complete filling zone, and the partial filling zone, listed from the bottom upward. To quantify the extent of the filling compaction zone, a dip-direction physical similar simulation experiment was conducted^25^. The backfill zone along the dip direction of the working face extends 40 m.

### Parameter selection and parameter calibration

The Mohr‑Coulomb constitutive model was selected to simulate the behavior of rock masses, which is widely adopted in geotechnical and mining engineering. For goaf backfilling, a double‑yield constitutive model was employed. Physical and mechanical parameters of the coal and rock strata were determined through field sampling and laboratory testing, as summarized in Table 1. In numerical simulations, directly applying laboratory‑measured rock mechanics parameters may lead to discrepancies between simulation results and actual engineering conditions, reducing the practical guidance value of the findings. Laboratory parameters typically represent small‑scale intact rock properties, while large‑scale numerical models must account for engineering‑scale rock mass behavior. Therefore, it is necessary to further optimize the parameters based on laboratory data. The Hoek–Brown criterion based on the GSI is commonly used to convert laboratory parameters into equivalent rock mass parameters^26–28^. Accordingly, the converted equivalent rock mass parameters, presented in Table 2, were applied in the numerical simulation.

**Table 1:** Coal and rock physical and mechanical parameters.

Number	Rock layers	Volume weight (kg/m^3^)	Modulus of elasticity (MPa)	Uniaxial compressive strength (MPa)	Cohesion (MPa)	Internal friction angle (°)
1	Siltstone	2350	5000	36.0	2.8	28
2	Coal	1350	3900	10.0	2.7	23
3	Carbon mudstone	2500	5500	18.0	4.0	23
4	Mudstone	2470	3800	20.0	2.9	30
5	Fine sandstone	2710	5600	34.0	3.9	25
6	Coarse sandstone	2570	4800	28.0	5.0	32
7	Sandstone	2520	4900	36.0	2.9	35
8	Fine sandstone	2710	5600	35.0	3.7	25
9	Conglomerate	2520	4900	29.0	3.0	33
10	Coarse sandstone	2570	4800	28.0	4.6	32
11	Backfill	1900	2900	/	0.1	20

**Table 2:** Rock mass characteristic parameters.

Number	Rock layers	Volume weight (kg/m^3^)	Modulus of elasticity (MPa)	Uniaxial compressive strength (MPa)
1	Siltstone	2350	102.5	2.24
2	Coal	1350	78.0	0.27
3	Carbon mudstone	2500	110.0	0.64
4	Mudstone	2470	76.0	0.94
5	Fine sandstone	2710	114.8	2.11
6	Coarse sandstone	2570	98.4	1.74
7	Sandstone	2520	100.4	2.24
8	Fine sandstone	2710	114.8	2.18
9	Conglomerate	2520	100.4	1.80
10	Coarse sandstone	2570	98.4	1.74

### Numerical simulation analysis process

The experimental procedure is as follows: first, the return airway and haulage roadway are excavated, each with a cross-sectional width and height of 5 m and 3 m, respectively. Normal mining then begins. According to the working face operation specifications, the caving interval is 1.2 m. Considering the block size in the numerical model, it was ultimately determined that after each 1 m advance of the working face, top-coal caving is performed at a position 5 m behind the face, with a caving interval of 1 m. The specific simulation process is as follows.

Step 1 (Coal Cutting): In each computational step, a strip of coal units is “excavated,” corresponding to the advance of one mining cycle.

Step 2 (Top-Coal Caving and Drawing): Caving Simulation: When a top-coal unit loses its underlying support (due to coal extraction) and its stress state satisfies the failure criterion (shear or tensile failure), the failed coal mass is simulated as fractured granular material. Drawing Simulation: In the designated “drawing zone” behind the working face, at each drawing step, a prescribed proportion of top-coal units in the “fractured state” are “removed” according to the field drawing practice—such as multi-round interval drawing or top-to-bottom sequencing—to simulate the outflow of fragmented coal through the drawing opening.

Each mining cycle advances by 1 m, and after each caving operation, the goaf is backfilled from the lower to the upper section along the dip direction of the working face, within a backfill range of 40 m. This process continues until mining is completed.

#### Layout of measuring surface

**Fig. 5:**
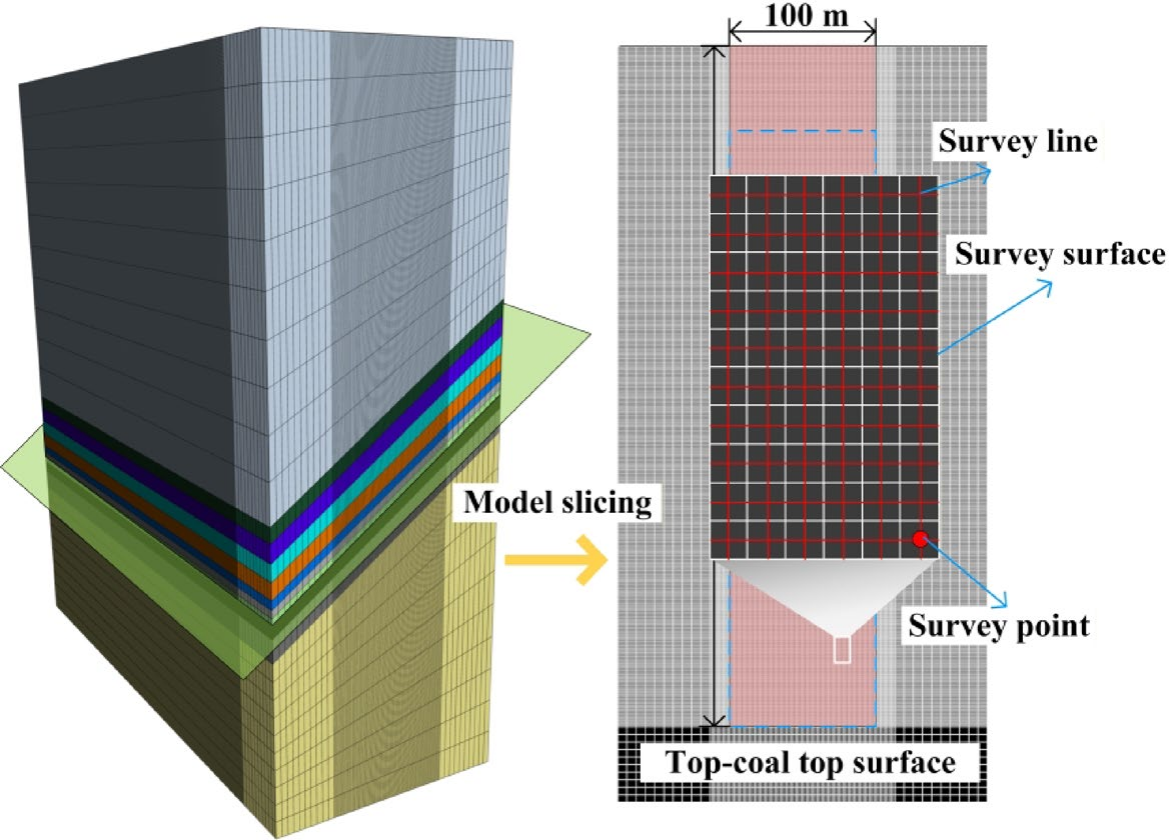
Layout of measuring surface of numerical model.

The focus of this study is the top-coal of the coal seam. To monitor the principal stress variations in different zones of the top-coal during numerical simulation, measuring surfaces were arranged as shown in Fig. 5. In the numerical model, a cross‑section was cut along the upper boundary of the top-coal to obtain a slice of this boundary. The red area in the figure defines the measuring surface. This surface measures 450 m in length and 100 m in width, fully covering the advancing region of the working face. By zooming in locally on the measuring surface, the positional relationships among measuring points, measuring lines, and surfaces can be clearly observed.

#### Extraction of principal stress data of top-coal

**Fig. 6:**
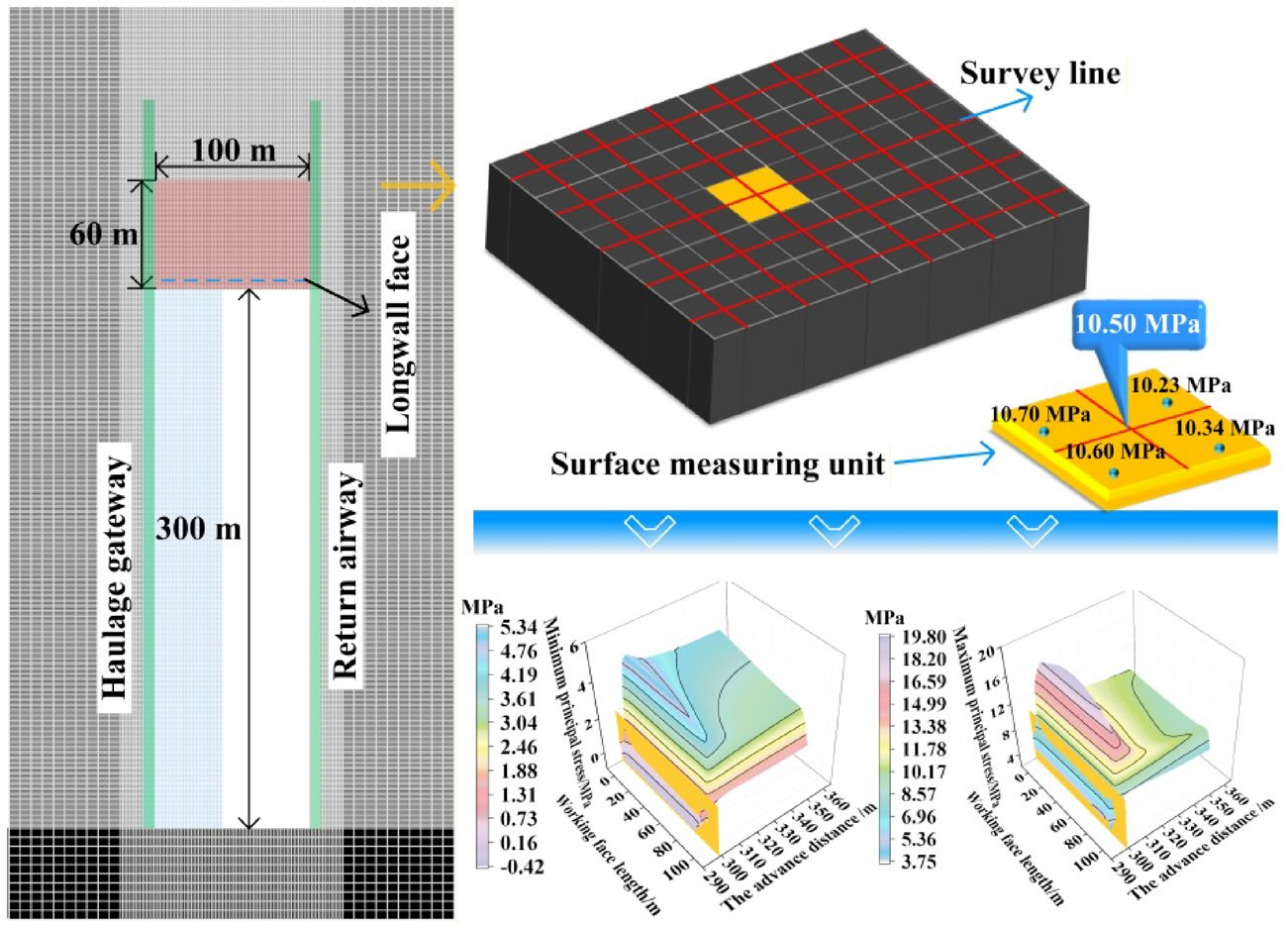
Extraction process of principal stress data.

The principal stress data of the top-coal were extracted from measuring surfaces arranged within the top-coal, as shown in Fig. 6. When the working face advanced to 300 m, principal stress data were collected from the measuring surfaces within a 60 m range of the top-coal. During extraction, since the top-coal was meshed with 1 m × 1 m square elements, the data obtained for each measuring surface unit represented the average value over the corresponding 1 m × 1 m area. In the figure, the blue numerical labels within each measuring surface unit denote these average values. The extracted data were then used to generate distribution maps of the principal stresses, yielding separate plots for the distribution patterns of the maximum and minimum principal stresses.

#### Realization of boundary spatial morphology of TLEZ

**Fig. 7:**
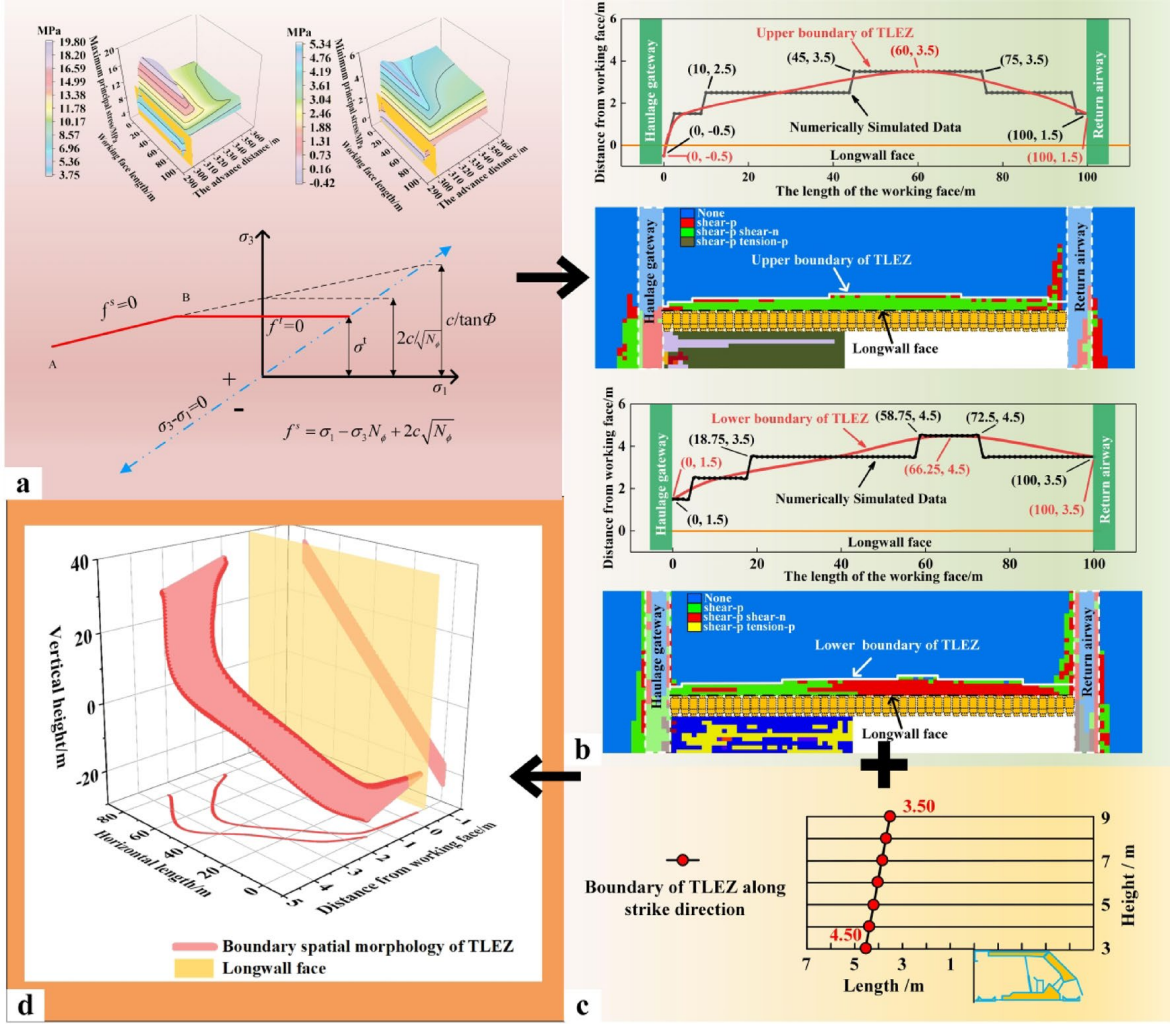
Quantization process of boundary spatial morphology of TLEZ.

The determination of the boundary spatial morphology in the TLEZ of a SDCS is achieved through a three‑step methodology. Step 1: Extraction of principal stress data along the upper and lower boundaries of the top-coal. As shown in Fig. 7a, the maximum and minimum principal stress data are extracted from the top-coal within a range of 60 m above the working face. Step 2: Determination of the distance from the upper and lower boundaries of the top-coal to the working‑face rib. The principal stress data and the composite Mohr-Coulomb criterion were integrated within the FLAC3D model. Using this approach, the positions of the upper and lower boundaries of the top-coal, relative to the working face rib, were identified, as shown in Fig. 7b. Step 3: Reconstruction of the spatial morphology of the limit equilibrium zone boundary. The positional relationships of the upper and lower boundaries of the limit equilibrium zone relative to the rib, combined with their distribution along the strike direction of the working face^29^, are synthesized to reconstruct the three‑dimensional boundary spatial morphology of the TLEZ, as shown in Fig. 7d.

## Evolution process of boundary spatial morphology of TLEZ in SDCS

### Evolution law of top-coal stress

In SDCS, influenced by geological structures, the in-situ stress varies along the dip direction of the working face. During the mining process, stress concentration forms ahead of the working face due to mining-induced effects. Consequently, affected by the dip angle of the coal seam, the abutment pressure ahead of the working face is the projection of each principal stress onto the normal direction of the coal seam plane. The stress state at any point within the rock mass can be determined using the principles of elasticity theory^25^. The critical transition point of mining-induced stress from the initial state to the stable state in the fully mechanized caving face was identified by analyzing the evolution of the front abutment pressure. Stress data associated with the advanced abutment pressure were extracted by instrumenting the upper boundary of top-coal with measurement faces. Based on this, the evolution patterns of the maximum and minimum principal stresses from the initial stage to the stable stage under mining-induced conditions were examined.

**Fig. 8:**
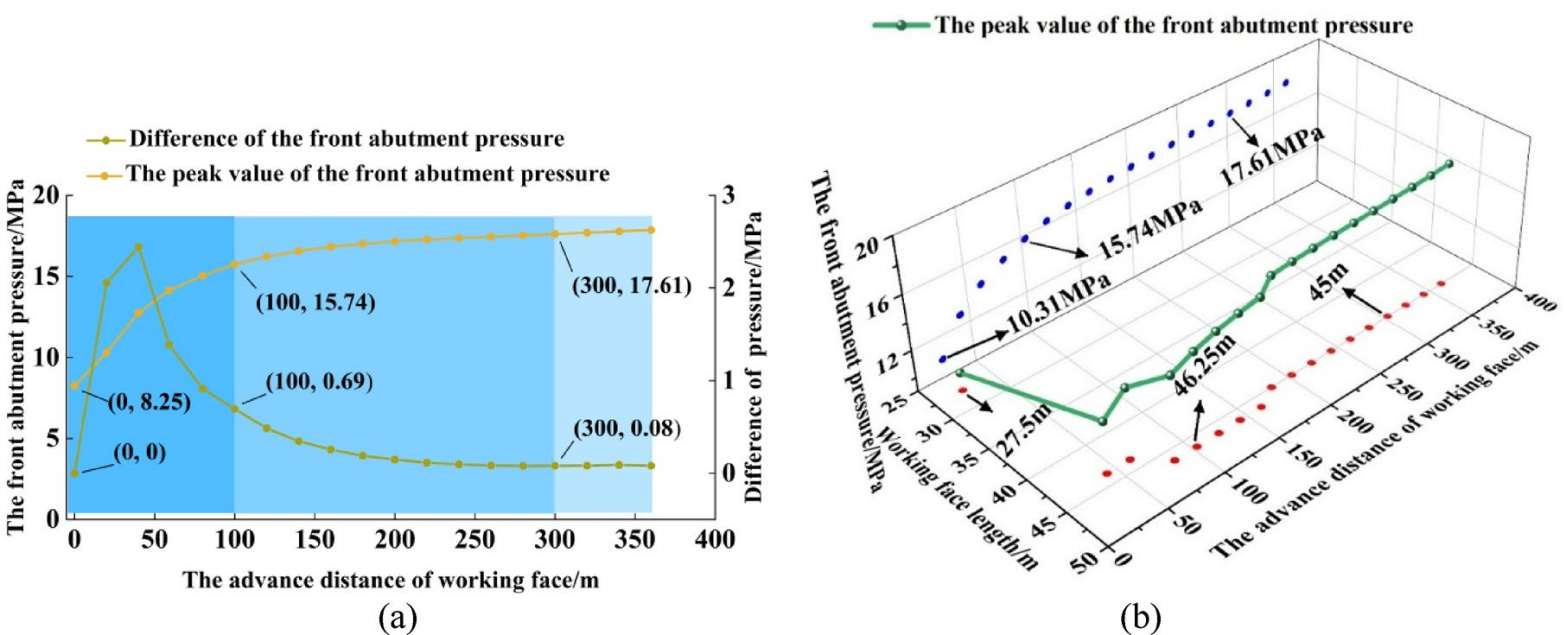
Spatial distribution law of peak value of advanced abutment pressure. (**a**) Peak evolution law (**b**) Evolution law of peak position.

Mining at the fully mechanized top-coal caving face progresses from an initial to a final stage. As mining advances, the mining-induced stress ahead of the working face evolves accordingly. This evolution can be divided into three distinct stages: growth, slow growth, and stable fluctuation. As shown in Fig. 8a, these stages correspond to the intervals of 0–100 m, 100–300 m, and 300–360 m, respectively. When the working face advanced from 0 m to 100 m, the peak stress increased from 8.25 MPa to 15.74 MPa, with a minimum stress difference of 0.69 MPa per 20‑m interval. As the face advanced from 100 m to 300 m, the peak stress rose from 15.74 MPa to 17.61 MPa, while the stress difference per 20‑m interval decreased from 0.69 MPa to 0.08 MPa. Beyond 300 m of advancement, the peak stress stabilized with minor fluctuations, and the stress difference per interval became nearly zero.

In the fully mechanized top‑coal caving face of SDCS, the location of the peak front abutment pressure differs significantly from that in horizontal seams. Due to the dip angle of the coal seam, as shown in Fig. 8b, the peak abutment pressure is not positioned at the middle of the working face, but shifts toward the lower part. As mining proceeds, the peak location gradually moves from the lower part toward the middle of the working face and eventually stabilizes at a position 45 m from the lower end of the face.

**Fig. 9:**
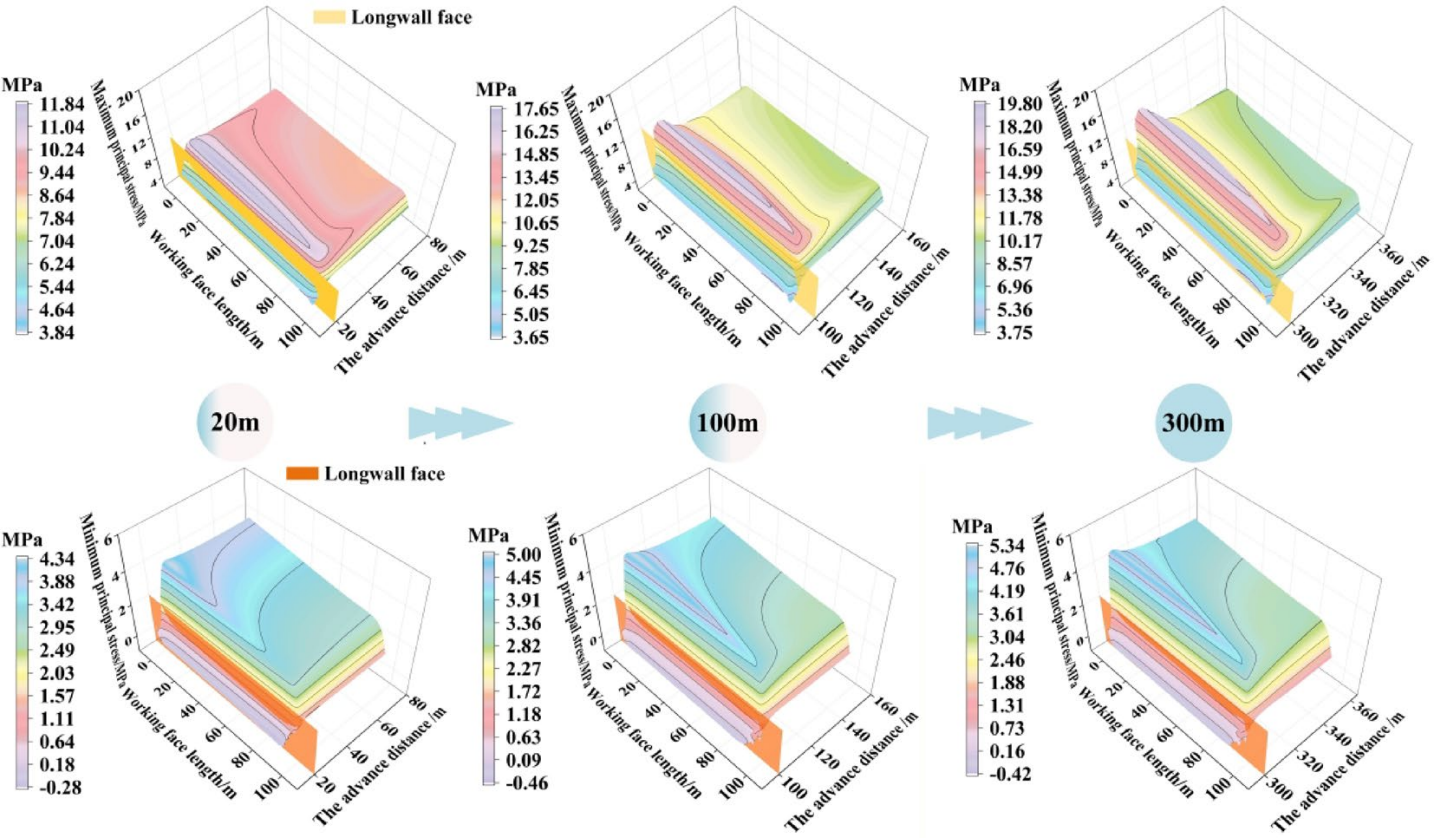
Distribution of maximum and minimum principal stress of top-coal under different advancing distances.

Based on the top‑coal stress data extraction scheme, distribution of the maximum and minimum principal stresses in the top-coal at different advancing distances of the working face were obtained, as shown in Fig. 9. Due to the gravitational-dip effect, the maximum principal stress along the dip direction of the working face shows the following pattern: the lower‑middle part > the upper‑middle part > the upper part > the lower part. The minimum principal stress exhibits a decreasing trend along the dip direction, with values following the order: the lower part > the lower‑middle part > the upper‑middle part > the upper part. Along the strike direction, both the maximum and minimum principal stresses follow a distribution pattern of increase–decrease–stabilization, with peak stresses located ahead of the working face. As the working face advances from 20 m to 100 m and finally to 300 m, the distribution patterns of the principal stresses remain unchanged, while their magnitudes increase with the intensity of mining activity.

### Evolution of boundary spatial morphology of TLEZ

**Fig. 10:**
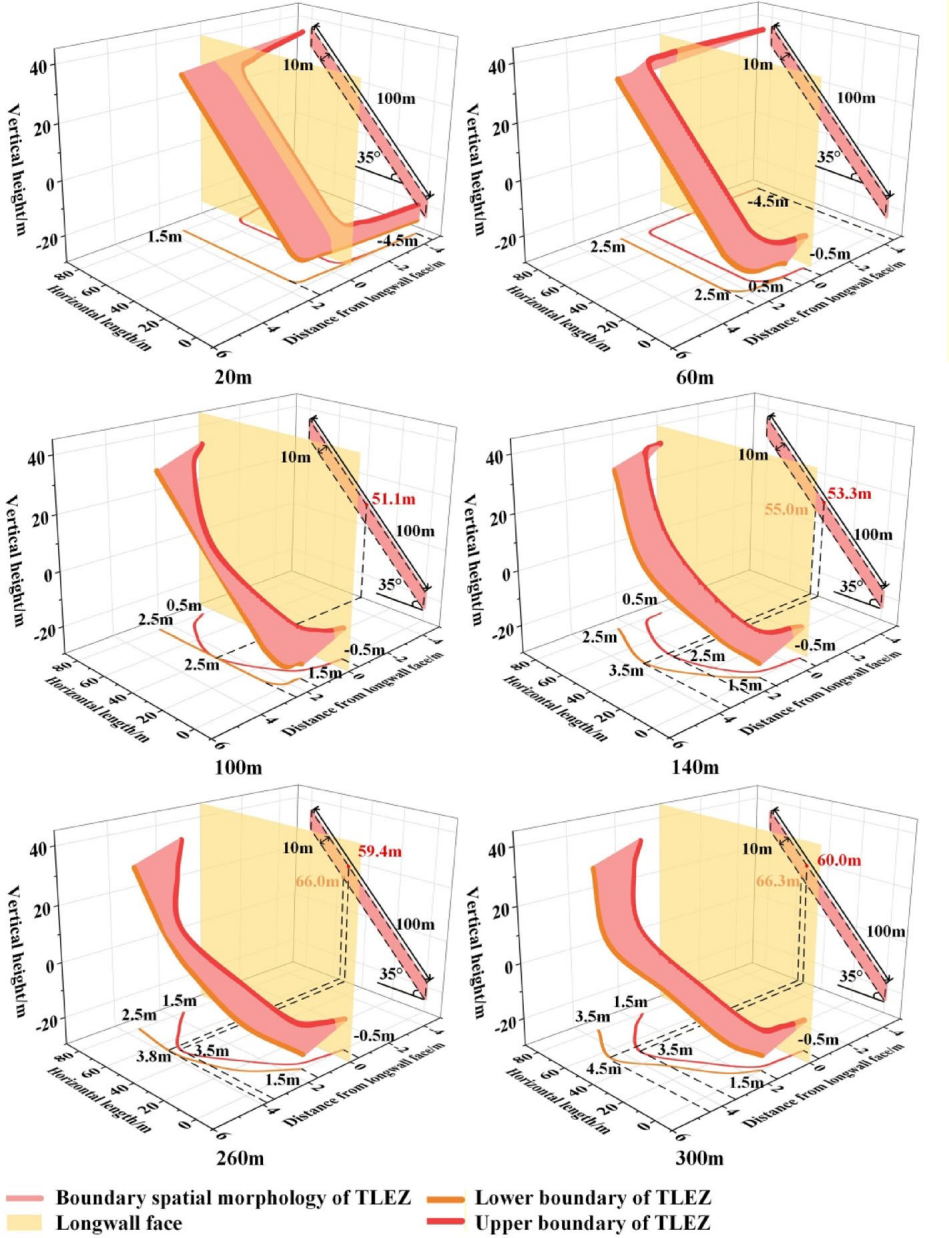
Evolution of boundary spatial morphology of TLEZ.

The boundary spatial morphology of the TLEZ is closely linked to the mining-induced stress in the working face, with each stage of mining resulting in a distinct morphological form. The transition from initial extraction to stabilized mining-induced stress constitutes a complex and dynamic process. As illustrated in Fig. 10, the evolution of the boundary spatial morphology in the TLEZ of SDCS demonstrates this progression: as the working face advances, the morphology evolves from an initial “irregular banded surface” into an “asymmetric arc-shaped ribbon-like curved surface.” This morphological evolution reflects the internal condition of the top-coal. Therefore, a clear understanding of its failure process and state at different stages is crucial for ensuring safe and efficient mining operations.

When the working face advanced to 20 m, the boundary spatial morphology appeared as an “irregular banded surface.” The lower boundary was generally located ahead of the upper boundary. Along the dip direction of the working face, the upper boundary of the TLEZ was situated behind the longwall face. At the 0 m position of the working face, both the upper and lower boundaries were 4.5 m behind the longwall face. Moving upward along the dip direction, the distance between the boundaries and the longwall face gradually decreased: the closest distance of the upper boundary to the longwall face was 0.5 m behind, while that of the lower boundary was 1.5 m ahead. Continuing upward along the dip direction, the distance of the upper boundary from the longwall face gradually increased, reaching 4.5 m behind the longwall face at the 100 m position, whereas the lower boundary remained unchanged.

When the working face advanced to 60 m, the boundary spatial morphology moved forward overall while maintaining its form. Along the dip direction at the 0 m position, the distance of the upper boundary from the longwall face decreased from 4.5 m behind to 0.5 m behind, and that of the lower boundary decreased from 4.5 m behind to 0.5 m ahead. Progressing upward along the dip, both boundaries continued to shift in the advancing direction, with the upper boundary reaching a maximum of 1.5 m ahead and the lower boundary 2.5 m ahead. Further upward along the dip, the distance of the upper boundary from the longwall face gradually reduced from 1.5 m ahead to 4.5 m behind.

When the working face advanced to 100 m, the upper boundary moved in the advancing direction and exhibited an asymmetric distribution. Along the dip direction at the 0 m position, the upper boundary remained unchanged, while the distance of the lower boundary from the longwall face increased from 0.5 m to 1.5 m. Progressing upward along the dip, the upper boundary continued to advance, and its distance from the longwall face gradually increased, reaching a maximum of 2.5 m at the 51.1 m position. Further upward along the dip, the distance decreased, returning to 0.5 m from the longwall face at the 100 m position. The lower boundary remained unchanged throughout this stage.

When the working face advanced to 140 m, the boundary spatial morphology of the TLEZ evolved from an “irregular banded surface” into an “asymmetric arc-shaped ribbon-like curved surface.” Along the dip direction at the 0 m position, both the upper and lower boundaries remained unchanged. Moving upward along the dip, the distance of the upper boundary from the longwall face stayed the same, but its position shifted toward the upper part of the working face, moving from 51.1 m to 53.3 m. Meanwhile, the distance of the lower boundary from the longwall face gradually increased, reaching a maximum of 3.5 m at the 55.0 m position. At the 100 m position, both boundaries remained unchanged.

When the working face advanced to 260 m, the boundary spatial morphology retained the shape of an “asymmetric arc‑shaped ribbon‑like curved surface” while continuing to move forward. Along the dip direction at the 0 m position, both the upper and lower boundaries remained unchanged. Progressing upward along the dip, the distances of both boundaries from the longwall face gradually increased. The upper boundary reached its maximum distance of 3.5 m at the 59.4 m position, while the lower boundary reached its maximum distance of 3.8 m at the 66.0 m position. At the 100 m position, the distance of the upper boundary from the longwall face increased from 0.5 m to 1.5 m, while the lower boundary remained unchanged.

When the working face advanced to 300 m, the boundary spatial morphology in the form of an “asymmetric arc‑shaped ribbon‑like curved surface” reached a stable state. Along the dip direction, the upper boundary remained unchanged. At the 0 m position of the working face, the lower boundary also remained the same. Moving upward along the dip, the position of the farthest distance of the lower boundary from the longwall face shifted upward from the 66.0 m position to the 66.3 m position, with the maximum distance increasing to 4.5 m. At the 100 m position, the distance of the lower boundary from the longwall face increased from 2.5 m to 3.5 m.

In summary, the evolution of the boundary spatial morphology in the TLEZ can be divided into three stages: the initial stage (0–140 m), the formation stage of the asymmetric arc‑shaped ribbon‑like curved surface (140–300 m), and the stable stage (300 m to the end of mining). Influenced by the dip angle of the coal seam, the evolution exhibits both asymmetry and sequentiality. Along the dip direction of the working face, the development proceeds successively from the lower, lower‑middle, and upper parts, and finally to the upper‑middle part. Along the strike direction, the evolution follows a top‑down order.

## Dip effect of the boundary spatial morphology in the TLEZ

SDCS are defined here as those with dip angles ranging from 35° to 55°. To investigate the dip effect on the boundary spatial morphology of the TLEZ, three specific dip angles—35°, 45°, and 55°—were selected for comparative analysis. In the numerical models, only the dip angle of the coal seam was varied, while all other parameters were held constant.

### Evolution of top-coal stress under different coal seam dip angle

**Fig. 11:**
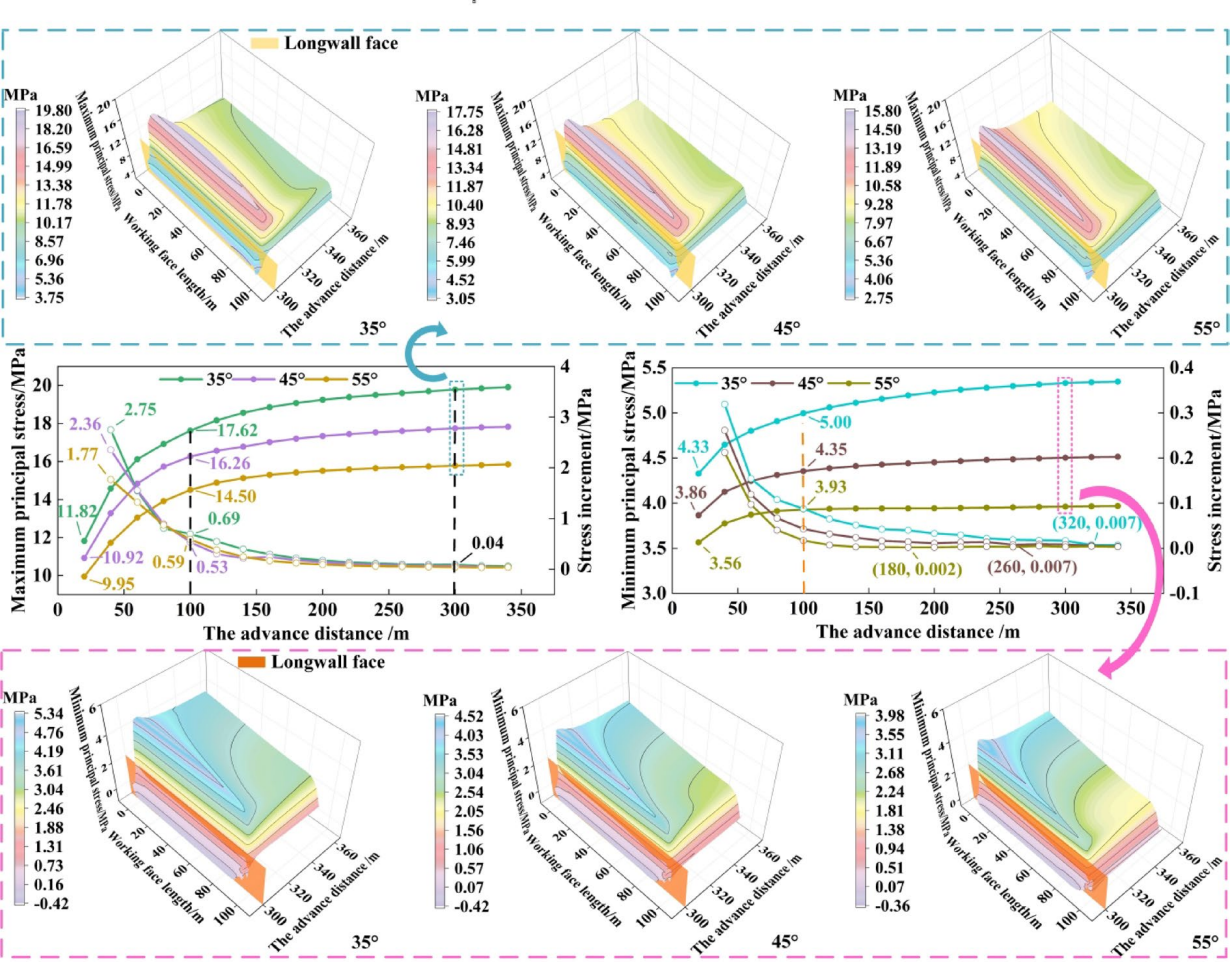
Evolution of principal stress under different coal seam dip angle.

Numerical simulations were conducted on fully mechanized caving faces in SDCS with varying dip angles. The distribution and evolution of mining-induced stress in the top-coal during extraction were determined. Based on these stress data, the boundary spatial morphology of the TLEZ at different mining stages was identified. Measuring surfaces were arranged along the upper boundary of the top-coal for dip angles of 35°, 45°, and 55°, each measuring 100 m in length and 60 m in width. By extracting the peak values of the maximum and minimum principal stresses, stress evolution diagrams were plotted, as shown in Fig. 11. In these diagrams, the left y-axis represents the peak stress magnitude, and the right y-axis indicates the peak stress difference per 20 m advance interval.

As the dip angle of the coal seam increases, the peak maximum principal stress decreases across different advancing distances. At 20 m of face advance, the peak stresses for dip angles of 35°, 45°, and 55° are 11.82 MPa, 10.92 MPa, and 9.95 MPa, respectively. At 100 m advance, the corresponding peaks are 17.62 MPa, 16.26 MPa, and 14.50 MPa. By 300 m advance, the peak stresses reach 19.80 MPa, 17.75 MPa, and 15.80 MPa, respectively. The growth rates of peak stress under different dip angles are similar as the face advances. As reflected by the slopes of the peak stress differences, the growth rates generally decline. During the mining stage from 20 m to 100 m, the stress differences for dip angles of 35°, 45°, and 55° decrease from 2.75 MPa, 2.36 MPa, and 1.77 MPa to 0.69 MPa, 0.59 MPa, and 0.53 MPa, respectively. After 300 m of advance, the stress difference further reduces to 0.04 MPa, with each stage exhibiting a decrease by roughly an order of magnitude.

As the dip angle of the coal seam increases, the peak values of the minimum principal stress decrease across all advancing distances. At 20 m of face advance, the peak minimum principal stresses for dip angles of 35°, 45°, and 55° are 4.33 MPa, 3.86 MPa, and 3.56 MPa, respectively. By 100 m advance, the corresponding peaks are 5.00 MPa, 4.35 MPa, and 3.93 MPa. At 300 m advance, the peak values reach 5.34 MPa, 4.52 MPa, and 3.98 MPa. The growth rates of the peak stress under different dip angles are similar as the face advances, with the slope of the peak stress difference indicating an overall declining trend consistent with the behavior of the maximum principal stress. Notably, as the dip angle increases, the peak minimum principal stress stabilizes earlier: for dip angles of 35°, 45°, and 55°, stress stabilization occurs at advancing distances of 180 m, 260 m, and 320 m, respectively.

Based on peak stress patterns, the stress evolution characteristics under different dip angles were evaluated. Three‑dimensional distribution maps of the maximum and minimum principal stresses at an advancing distance of 300 m are shown in Fig. 11 for various dip angles. The principal stress distributions follow consistent trends along both the dip and strike directions. As the dip angle increases, the peak regions of the maximum and minimum principal stresses shift toward the lower part of the working face, and stress asymmetry along the dip direction becomes more pronounced. This asymmetry alters the progressive degradation trend of the top- coal, leading to changes in the boundary spatial morphology of the TLEZ, thereby affecting the safety and efficiency of mining operations in fully mechanized top‑coal caving faces in SDCS.

### Dip effect on the evolution of boundary spatial morphology in the TLEZ

The boundary spatial morphology of the TLEZ in SDCS is influenced by the in‑situ stress environment and the mining‑induced stress from the working face. Under different coal seam dip angle, the stress conditions acting on the seam vary, which directly governs the failure process of the top-coal. This failure process, in turn, determines the evolution of the boundary spatial morphology of the TLEZ. Conversely, determining the boundary spatial morphology of the TLEZ allows for an indirect assessment of the coal seam failure process. Based on this morphology, on‑site adjustments can be made to continuously ensure safe and efficient mining operations at the working face.

**Fig. 12:**
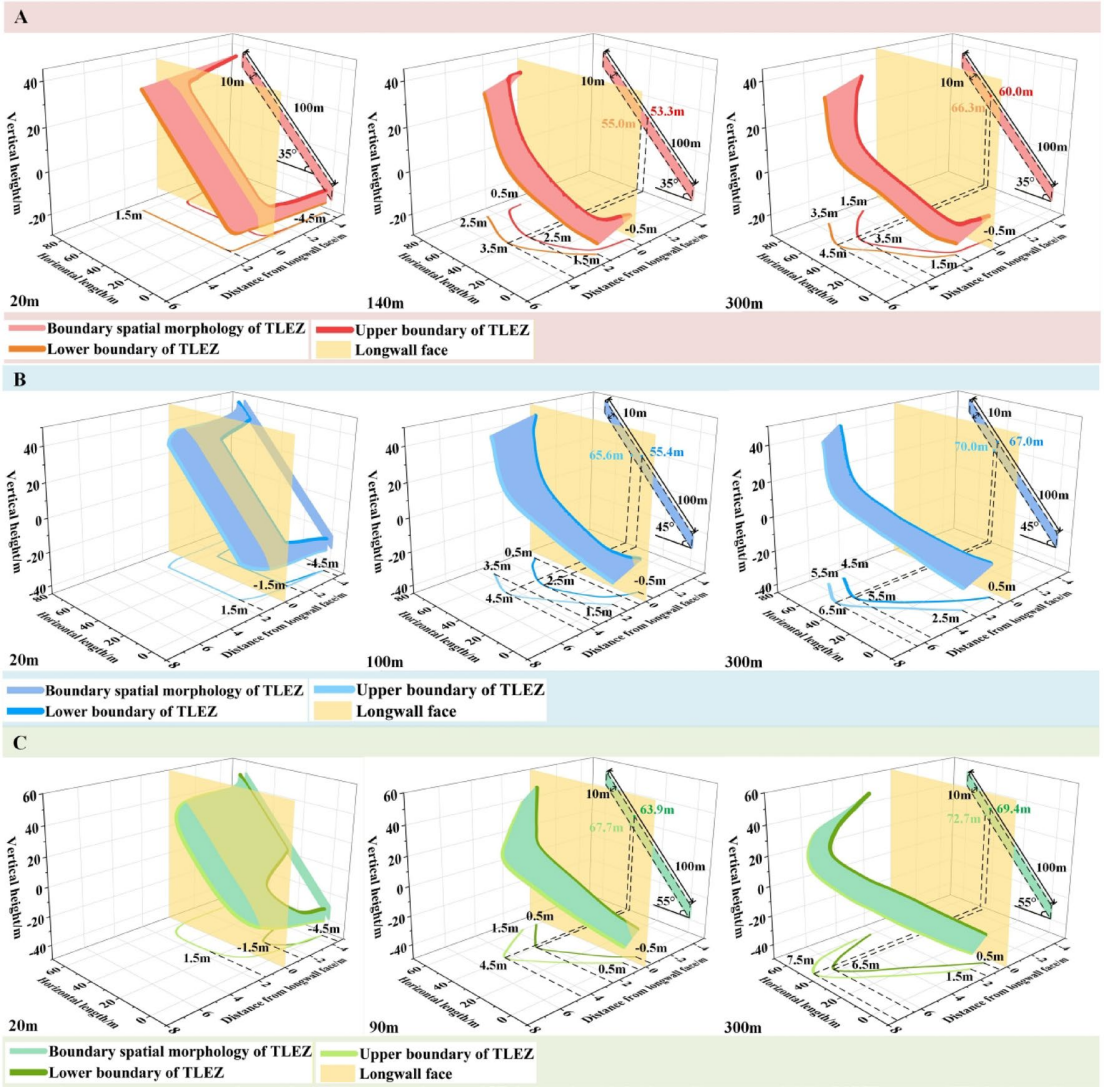
Evolution of the boundary spatial morphology of top-coal under different coal seam dip angle.

The boundary spatial morphologies at different stages for coal seam dip angles of 35°, 45°, and 55° are shown in Fig. 12, corresponding to the initial stage, the formation stage of the “asymmetric arc‑shaped ribbon‑like curved surface”, and the stable stage, respectively.

Initial stage. When the working face advanced to 20 m, the boundary spatial morphology generally exhibited an “irregular twisted band.” During the early mining phase, the influence range of mining‑induced stress was limited. The entire upper boundary was located behind the longwall face, while part of the lower boundary extended ahead of it. The boundaries were farthest from the longwall face near both sides of the roadway. The mining effect from the working face was greater than that from the roadway excavation. As the dip angle of the coal seam increased, the distance between the upper and lower boundaries along the strike direction gradually widened.

Formation stage of the “asymmetric arc-shaped ribbon-like curved surface”. With increasing coal seam dip angle, when the dip angles are 35°, 45°, and 55°, the boundary spatial morphology of the TLEZ evolves from an “irregular twisted band” to an “asymmetric arc-shaped ribbon-like curved surface” at advancing distances of 140 m, 100 m, and 90 m, respectively. The asymmetry of the boundary spatial morphology is skewed toward the upper-middle part of the working face, where the distance to the longwall face is greatest. From this central upper region, the distance to the longwall face gradually decreases both downward and upward along the dip direction. At the 0 m position of the working face, the distances of the upper and lower boundaries from the longwall face for dip angles of 35°, 45°, and 55° are − 0.5 m and 1.5 m, − 0.5 m and 1.5 m, and − 0.5 m and 0.5 m, respectively. At the 100 m position, the corresponding distances are 0.5 m and 2.5 m, 0.5 m and 3.5 m, and 0.5 m and 1.5 m. Moving from top to bottom along the strike direction, the asymmetric vertex progressively shifts toward the upper part of the working face along the dip direction. For dip angles of 35°, 45°, and 55°, the asymmetric vertices of the upper and lower boundaries are located at 53.3 m and 55.0 m, 55.4 m and 65.6 m, and 63.9 m and 67.7 m along the working face, respectively. At these vertices, the distances to the longwall face are greatest: 2.5 m and 3.5 m, 2.5 m and 4.5 m, and 2.5 m and 4.5 m, respectively. As the dip angle rises, the asymmetric distribution of the boundary spatial morphology shifts further upward along the dip direction, the distance from the longwall face increases, and the influence range of mining-induced stress expands correspondingly.

Stable stage. At an advancing distance of 300 m, mining‑induced stress under different coal seam dip angle stabilized, resulting in a corresponding stabilization of the boundary spatial morphology of the TLEZ. In this stable state, the boundary spatial morphology displays two clear trends with increasing dip angle: First, the extent of top‑coal failure progressively expands. At the 0 m position along the working face, the distances of the upper and lower boundaries from the longwall face for dip angles of 35°, 45°, and 55° are − 0.5 m and 1.5 m; 0.5 m and 2.5 m; and 0.5 m and 1.5 m, respectively. At the 100 m position, these distances increase to 1.5 m and 3.5 m, 4.5 m and 5.5 m, and 2.5 m and 3.5 m. For dip angles of 35°, 45°, and 55°, the distances of the asymmetric vertices of the upper and lower boundaries from the longwall face are 3.5 m and 4.5 m, 5.5 m and 6.5 m, and 6.5 m and 7.5 m, respectively. With increasing dip angle, both the volume of top-coal within the boundary spatial morphology and the failure depth increase. The maximum failure depths at dip angles of 35°, 45°, and 55° are 4.5 m, 6.5 m, and 7.5 m, respectively. Second, asymmetry becomes more distinct. As the dip angle increases, the vertices of the upper and lower boundaries for dip angles of 35°, 45°, and 55° are located at 60.0 m and 66.3 m, 67.0 m and 70.0 m, and 69.4 m and 72.7 m along the working face, respectively. The asymmetric region of the boundary spatial morphology shifts from the upper-middle part toward the upper part of the working face along the dip direction.

## Discussion

### Relationship between boundary spatial morphology of TLEZ and stability of the “support surrounding rock” system

Acting as the only medium between the support and the roof, the load transfer capability of top-coal decisively influences the effectiveness of the “support-surrounding rock” system in SDCS. When top-coal is less fragmented, its load carrying capacity is higher; conversely, greater fragmentation reduces this capacity. Under mining-induced stress, top-coal undergoes a complex loading process, transitioning from an initial elastic state to a limit equilibrium state and finally to a granular state before being discharged through the drawing opening.

The failure of the coal mass occurs as top-coal moves from the elastic to the limit equilibrium state. During the subsequent transition from the limit equilibrium state to the granular state, broken coal blocks continue to exert confinement on adjacent material, and interactions among fragments allow the damaged coal to retain a certain load bearing capacity. Consequently, the damaged coal must sustain the load acting from the boundary of the limit equilibrium zone until it is released at the drawing opening. The longer the damaged coal remains under load before discharge, the greater its degree of fragmentation. This loading duration is directly linked to the boundary spatial morphology of the TLEZ.

**Fig. 13:**
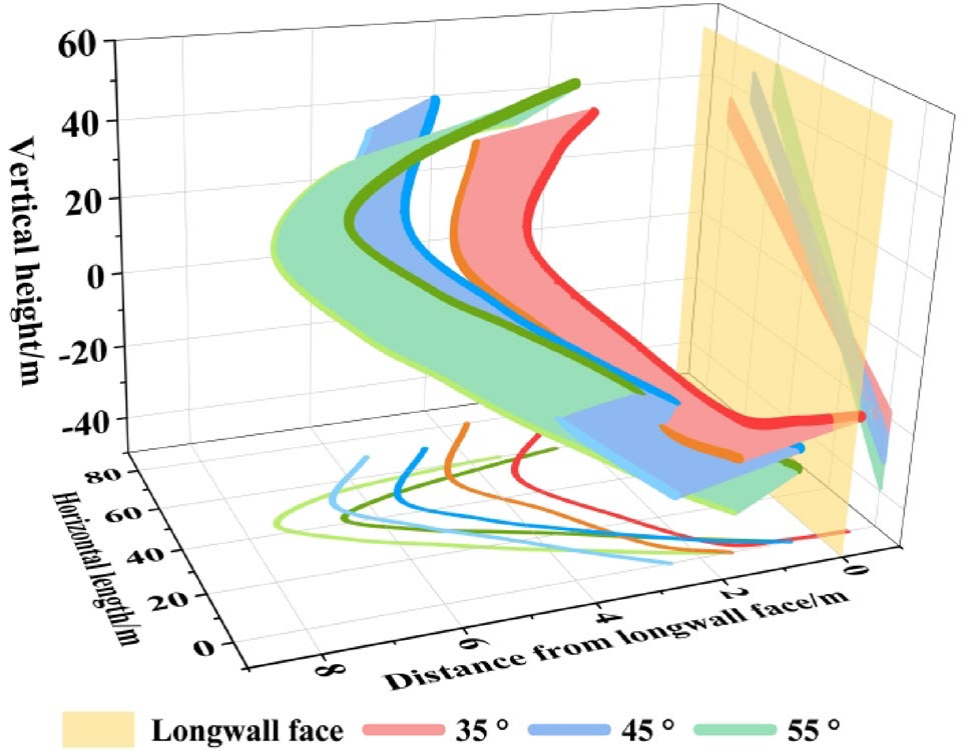
Boundary spatial morphology of the TLEZ under different coal seam dip angle.

During the fully mechanized top‑coal caving process in SDCS, the dip effect leads to an “asymmetric distribution” of the TLEZ boundary along the dip direction of the working face. Taking the boundary spatial morphology of the TLEZ under dip angles of 35°, 45°, and 55° as examples, as shown in Fig. 13, the upper‑middle part of the working face exhibits the largest distance to the longwall face, followed by the upper part and the lower‑middle part, while the lower part of the working face shows the shortest distance. The distance from the TLEZ boundary to the longwall face is positively correlated with the fragmentation degree of the top-coal: the farther the boundary is from the face, the more severe the top‑coal fragmentation.

**Fig. 14:**
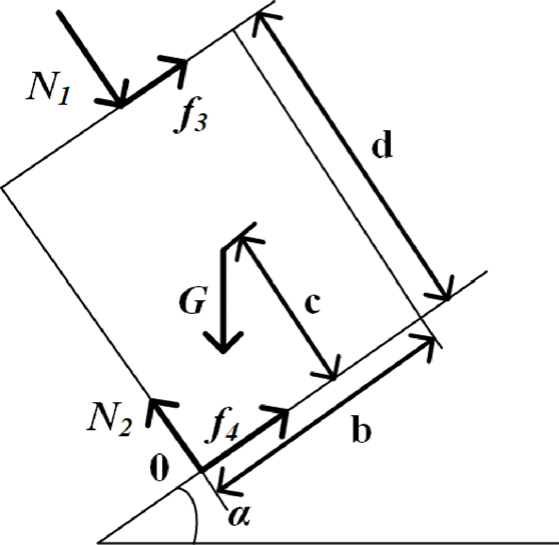
Mechanical model of critical collapse of support.

The degree of top-coal fragmentation directly affects coal caving performance and support behavior at the working face. When the coal above the support is highly fragmented, its load transfer capacity decreases, which can lead to support slippage and toppling. To investigate the relationship between support stability and top-coal fragmentation, a mechanical model for a single support under toppling tendency in fully mechanized caving face of SDCS was established^30^, as shown in Fig. 14. In such a face, when a support tends to topple, it is subjected not only to its own weight, but also to the friction forces exerted by the top-coal on the canopy, the friction forces from the floor on the base, the load transmitted from the top-coal, and the supporting reaction from the floor.

In a fully mechanized top-coal caving face with a coal-seam dip angle of α, the resultant force acting on a single support when it tends to topple passes through point 0, which is located at the lower edge of the support base. Taking point 0 as the center, the moment equilibrium equation for the support under toppling tendency can be expressed as follows:

1 $$Gc\sin \alpha = G\cos \alpha \frac{b}{2} + N_{1} \frac{b}{2} + f_{3} d$$

Where *N*_1_ is the pressure on the support canopy (N), *f*_3_ is the friction force on the canopy (N), *α* is the dip angle of the coal seam, c is the distance from the support’s center of gravity to the floor (m), d is the height of the support (m), b is the width of the support (m).

From Eq. (1), the condition for a single support to start toppling can be derived as:

2 $$Gc\sin \alpha > G\cos \alpha \frac{b}{2} + N_{1} \frac{b}{2} + f_{3} d$$

According to Eq. (2), while parameters such as the self weight of the support, base width, height of the center of gravity, and coal seam dip angle are all known, the decisive factors affecting support toppling are the pressure on the canopy and the friction force acting on it. If these two forces are too small, the support becomes highly prone to instability. The magnitudes of these forces are closely linked to the degree of fragmentation of the top-coal above the support. The more severe the fragmentation of the top-coal, the more significantly its load‑transfer capacity decreases. This reduction also leads to a decrease in the friction force acting on the canopy, thereby weakening the stability of the support and ultimately compromising the “support‑surrounding rock” system.

Field monitoring was conducted at the 1123 fully mechanized top-coal caving face, during which the frequencies of top-coal leakage and the support resistance were recorded. The frequency of top-coal leakage reflects the degree of coal fragmentation, while the support resistance indicates the stability of the supports. By analyzing the distribution of the distance from the lower boundary of TLEZ to the working face, the frequency of top-coal leakage, and the support resistance, the relationship between the TLEZ boundary distance, the degree of top-coal fragmentation, and support stability can be quantitatively correlated. Furthermore, the accuracy of the numerical simulation results was validated.

**Fig. 15:**
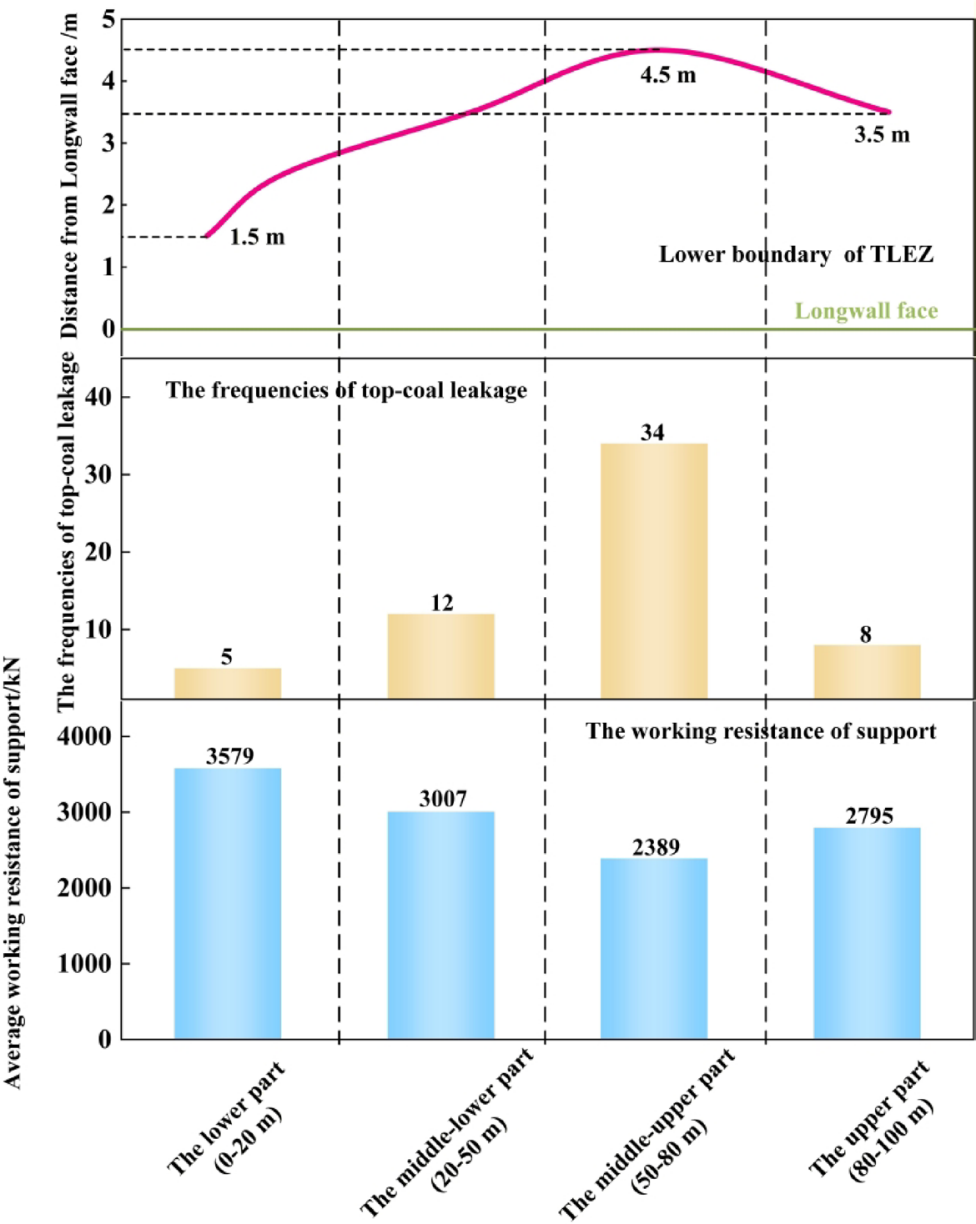
Distribution of the frequencies of top-coal leakage and working resistance of support.

As shown in Fig. 15, in the middle-upper part of the working face, the lower boundary of TLEZ was furthest from the longwall face at 4.5 m, the frequency of top-coal leakage was highest at 34 occurrences, and the support resistance was lowest at 2,389 kN. In the lower part of the working face, the lower boundary of TLEZ was closest to the longwall face at 1.5 m, the frequency of top-coal leakage was lowest at 5 occurrences, and the support resistance was highest at 3,579 kN. These monitoring results confirm the relationship between the distance from the boundary of TLEZ to the working face, the degree of top-coal fragmentation, and support stability. Therefore, by investigating the evolution process of the boundary spatial morphology of the TLEZ and its dip effect, a connection has been established among the distance from the boundary spatial morphology of the TLEZ to the working face, the degree of top‑coal fragmentation, and the stability of the “support‑surrounding rock” system. This linkage provides targeted guidance for the layout and control of working faces in similar mining conditions.

### Limitations of numerical simulation

Coal seam formation is a complex process that involves the transformation of plant remains through peatification, diagenesis, and metamorphism, ultimately resulting in coal. Consequently, before mining, the combination of structural bodies and structural planes within a coal seam is inherently intricate. This complexity increases further with greater coal seam thickness. In the numerical modeling process, the rock mass is typically assumed to be homogeneous and isotropic, without considering the influence of actual structural features on its mechanical behavior. To mitigate the deviation caused by this assumption, this study converts laboratory‑measured rock physico‑mechanical parameters into equivalent rock mass parameters using the GSI‑based Hoek‑Brown criterion, aiming to approximate real engineering conditions as closely as possible. However, regardless of how parameters are adjusted, inevitable discrepancies remain between numerical simulation results and actual field conditions. Despite this, the qualitative analysis conducted in this study on the evolution of the boundary spatial morphology of the top‑coal limit equilibrium zone and its dip effect yields conclusions that are universally applicable and do not change with specific engineering backgrounds. These findings can provide theoretical support for the layout and surrounding rock control of working faces under similar geological and mining conditions, thereby contributing to safe and efficient production.

## Conclusion

1. As the dip angle of the coal seam increases, the peak values of both the maximum and minimum principal stresses decrease. Along the strike direction of the working face exhibit a distribution pattern of “increase–decrease–stabilization.” Along the dip direction, the maximum principal stress shows a trend where the middle part > upper part > lower part of the working face, while the minimum principal stress follows the order: lower part > middle part > upper part.
2. The evolution of the boundary spatial morphology of the TLEZ in SDCS can be divided into three stages: the initial stage, the formation stage of the “asymmetric arc‑shaped ribbon‑like curved surface,” and the stable stage. Along the dip direction, the boundary morphology exhibits asymmetry, and the degree of asymmetry gradually intensifies as the working face advances. Along the dip direction of the working face, the evolution occurs sequentially from the lower, lower-middle, upper, to the upper-middle parts. Along the strike direction, the evolution proceeds in a top-down order.
3. With the increase in coal seam dip angle, the evolution rate of the boundary spatial morphology accelerates. The failure range of the top-coal expands, and the volume of top-coal encompassed within the boundary spatial morphology progressively increases. The asymmetry of the boundary spatial morphology becomes more pronounced.
4. The relationship between the boundary spatial morphology of the zone, the degree of top‑coal fragmentation, and the stability of the “support‑surrounding rock” system is established. The farther the boundary spatial morphology is from the longwall face, the higher the degree of top‑coal fragmentation, the more prone the working face support is to instability, and the lower the stability of the “support‑surrounding rock” system.

## Data Availability

The data that support the findings of this study are available from the corresponding author upon reasonable request.
